# Evaluation of Knowledge of Human Papillomavirus Infection and Its Oral Health Implications: A Comparative Study of Polish Medical and Dental Students

**DOI:** 10.3390/jcm14082695

**Published:** 2025-04-15

**Authors:** Paulina Adamska, Natalia Kempa, Dominika Cichońska, Adam Zedler, Hanna Sobczak-Zagalska

**Affiliations:** 1 Division of Oral Surgery, Medical University of Gdańsk, 7 Dębinki Street, 80-211 Gdansk, Poland; adam.zedler@gumed.edu.pl; 2 Scientific Circle of Oral Surgery, Medical University of Gdańsk, 7 Dębinki Street, 80-211 Gdansk, Poland; natalia.kempa@gumed.edu.pl; 3 Department of Periodontology and Oral Mucosa Diseases, Medical University of Gdańsk, 18 Orzeszkowej Street, 80-204 Gdansk, Poland; dcichonska@gumed.edu.pl; 4 Department of Pediatric Dentistry, Medical University of Gdansk, 18 Orzeszkowej Street, 80-204 Gdansk, Poland; h.zagalska@gumed.edu.pl

**Keywords:** HPV, human papillomavirus, knowledge, mouth diseases, oral cancer, papillomavirus infections, surveys and questionnaires

## Abstract

**Background:** Human papillomavirus (HPV) is a virus belonging to the *Papillomaviridae* family. Infection may be asymptomatic, mildly symptomatic, or clinically evident. It is mostly transmitted through sexual activity. It is estimated that approximately half of sexually active individuals will be infected with HPV, and among women over 50, this percentage increases to 80%. The aim of this study was to assess the level of awareness among medical and dental students regarding the impact of HPV infection on oral health. **Materials and methods:** This was a prospective study of 562 Polish medical and dental students. The questionnaire was originally designed based on the available literature and consisted of two sections: demographic questions and detailed questions about HPV and its impact on oral health. The data were analyzed using Statistica v. 13.3. All tests were considered statistically significant at *p* ≤ 0.05. **Results:** After excluding incomplete questionnaires, 541 remained and were included in the analysis. The medical students achieved a significantly higher average score compared to the dental students (*p* < 0.001). The students of higher years of studies were more knowledgeable. This tendency was observed both among all respondents together and among the medical and dentistry students separately. **Conclusions:** The medical students demonstrated a significantly higher level of knowledge on the discussed topic. However, both groups provided correct answers at a relatively unsatisfactory level because the overall value of the results obtained was below 60%. By the end of their education, the students in both fields demonstrated improved knowledge. The dental students gave an average of 74% correct answers, while the medical students achieved 73%. Therefore, the quality of university education regarding the relationship between HPV infection and oral lesions should be improved.

## 1. Introduction

Human papillomavirus (HPV) belongs to the *Papillomaviridae* family and is a deoxyribonucleic acid (DNA) virus [1]. It was first described in 1949 by Strauss [2]. The HPV genome consists of three distinct regions. The virus particle has a diameter of 50–60 nm and is enclosed by 72 protein capsomeres. It is a non-enveloped virus. HPV can be classified into low-risk (LR-HPV) and high-risk (HR-HPV) types [1,3].

Viruses from the *Papillomaviridae* family have developed adaptive mechanisms that enable them to evade the body’s immune response. To date, more than 448 HPV genotypes have been identified, with 12 classified as carcinogenic, i.e., types 16, 18, 31, 33, 35, 39, 45, 51, 52, 56, 58, and 59, with types 16 and 18 being the most prevalent [4]. Approximately 80% of infections caused by oncogenic HPV types are cleared by the host’s immune system, with only a small percentage progressing to a precancerous or cancerous state [4,5]. The remaining infections do not lead to cancer. HPV primarily targets differentiating squamous epithelium, causing skin and mucosal lesions [1,4,6]. The morphology and capsid structure of HPV are consistent across the *Papillomaviridae* family. Different HPV groups are distinguished based on variations in amino acid sequences and genome organization [1,4,5,6].

The exact mechanism of HPV infection is not yet fully understood. The most widely accepted theory suggests that HPV enters host cells through micro-injuries in the basement membrane of the epithelium. The virus gains entry via endocytosis and is subsequently transported to the nucleus, where it undergoes replication and transcription. The HPV genome consists of eight open reading frames: *E1*, *E2*, *E4*, *E5*, *E6*, and *E7*—with *E6* and *E7* playing a crucial role in disrupting host cell growth and differentiation—as well as two late regions, *L1* and *L2* [2,4,5].

HPV is primarily transmitted through sexual contact and is the most common sexually transmitted infection worldwide [2,7,8]. Other routes of transmission include direct skin-to-skin or skin-to-mucosa contact. Less frequently, the virus can spread perinatally. In extremely rare cases, auto-inoculation or indirect transmission has been reported in individuals with no history of sexual intercourse [2].

HPV infection may be asymptomatic, mildly symptomatic, or clinically apparent. It can cause lesions on various parts of the body or superinfection of existing ones. These lesions may affect the feet, hands, and genital area, and in some cases, HPV infection can lead to cancer [2,4]. HPV is responsible for approximately 4.5% of all human cancers. Oncogenic subtypes of the virus contribute to the development of several malignancies, including cervical, vaginal, vulvar, and penile cancer, as well as head and neck cancers (*oropharyngeal*, *laryngeal*, *and oral cancer*), esophageal cancer, brain cancer (*glioblastoma multiforme*), and lung cancer [5].

Globally, approximately 450,000 cases of oropharyngeal squamous cell carcinoma (OPSCC) and lip cancer are diagnosed annually, with 30% of cases linked to HPV infection [6]. The incidence of OPSCC is among the fastest-growing cancers in Europe and worldwide. One of the most significant risk factors for head and neck squamous cell carcinoma (HNSCC), particularly OPSCC, is alcohol and tobacco consumption. However, in recent decades, oncogenic HPV infection has emerged as an equally critical risk factor. This association was first described in 1983 [3,6]. Additionally, a higher number of lifetime oral sex partners is a recognized risk factor for HPV-related OPSCC. HPV-related squamous cell carcinoma of the oropharynx (HPV+ OPSCC) accounts for approximately 33% of OPSCC cases worldwide as of 2021. Among OPSCC cases, HPV16 is the dominant genotype, responsible for 86% of HPV+ OPSCC cases. Other high-risk HPV (HR-HPV) genotypes include HPV 18, 26, 31, 33, 35, 45, 56, 58, 59, and 67 [3,6].

Cure rates for HPV-positive OPSCC are higher than those for tobacco- and alcohol-related OPSCC [6]. Early diagnosis plays a crucial role in determining a patient’s prognosis, with HPV status being one of the most significant prognostic factors. Therefore, HPV testing is an important tool for estimating prognosis. A significantly worse prognosis is observed in a subgroup of patients with HPV subtype 16. Additionally, clinical prognosis is influenced by the location and size of the primary lesion, lymph node involvement, presence of positive surgical margins, and degree of tumor differentiation (well, moderately, or poorly differentiated) [6]. Other prognostic factors include lymphatic invasion and perineural invasion. The recurrence rate in patients with HPV-positive OPSCC is approximately 10–25% within 2–5 years of the initial diagnosis. Close monitoring of patients in remission is essential. The five-year survival rate for HPV-associated OPSCC is around 80%, whereas the overall survival rate for this disease typically ranges from 25% to 50% [6]. The high mortality rate associated with oral cancer is largely due to the late-stage diagnosis in a significant proportion of patients. For this reason, both moderate- and high-risk patients should undergo regular oral and throat examinations, with a particular focus on oncological prevention. Patients should also be encouraged to perform oral self-examinations [6,9].

Medical and dental students, as future practitioners, should have basic knowledge about the diagnosis and treatment of HPV-related conditions. Diseases induced by HPV infection and their complications require the involvement of many specialists and can lead to serious health complications. In Poland, medical doctors undergo a six-year education program, while dentists undergo a five-year education program, followed by a one-year postgraduate internship. During their studies, students are introduced to HPV-related topics in courses such as microbiology, dermatology and venereology, infectious diseases, oncology, epidemiology, gynecology, laryngology (for both fields of study), mucosal diseases, oral surgery, periodontology, and maxillofacial surgery (for dental students). The first information appears already in the first year of studies.

Late detection of head and neck cancer is associated with significant health, social, and economic costs. Treatment initiated at advanced stages is associated with lower efficacy or limited to palliative care. Early detection of HPV-related head and neck cancers is associated with a better prognosis compared to tobacco-related cancers [6,10,11,12]. Patients often experience mental health disorders due to changes in appearance caused by the location of the disease. Moreover, advanced stages generate higher healthcare costs and social consequences, as they often remove patients from the job market.

Poland is among the countries with relatively high morbidity and mortality of oral and oropharyngeal cancers. Cervical cancer is also a significant issue, with a mortality rate twice as high as the European Union average [13]. This is concerning, as preventive measures—such as regular check-ups, Pap smears, and vaccinations—are highly effective. However, Poles are not inclined to attend follow-up visits, and the vaccination rates among children remain alarmingly low [10,11,12]. Only two-thirds of Polish women visit a gynecologist for an annual check-up [14], while just over half of Poles see a dentist once a year or every six months [15]. In 2019, HPV vaccination coverage was below 10% among the population eligible for vaccination. After HPV vaccination was included in the recommended immunization program in 2023, coverage increased to approximately 18.3% of eligible adolescents [10,11,12]. Therefore, health promotion and the education of medical personnel are crucial.

The aim of this study was to assess the level of awareness among medical and dental students of the impact of HPV infection on oral health.

## 2. Materials and Methods

A prospective study was conducted on 562 Polish dental and medical students. The study participants were recruited between April and November 2020. The survey was distributed via email to 12 Polish medical universities, among which 10 had both medical and dental faculty. It was also shared on social media platforms within student associations. All the participants provided informed consent to participate in this study. Full anonymity was maintained. This research was approved by the institutional ethics committee (Independent Bioethics Commission for Research, Medical University of Gdańsk, approval number NKBBN/383/2020). All the participants were informed about the use of their data for research purposes.

### 2.1. Inclusion and Exclusion Criteria

The inclusion criteria were as follows: Polish-speaking students enrolled in dentistry or medicine at Polish universities who fully completed the questionnaire.

The exclusion criteria were as follows: individuals with limited proficiency in Polish, non-dental or non-medical students, and those who submitted an incomplete questionnaire.

### 2.2. Questionnaire

The questionnaire was prepared originally, was based on the available literature, and consisted of two parts: (I) basic information (six questions) and (II) HPV-specific questions (20 questions). Completion of the questionnaire took about 15 min. Among the questions about HPV infection, 17 questions were single-choice and 3 were multiple-choice. The survey results were also summarized collectively. One point was awarded for each correct answer to the single-choice questions, while zero points were given for incorrect answers. A different scoring method was applied to the multiple-choice questions. To receive one point for each of these three questions, the respondent had to select at least 50% of the correct answers. The minimum number of points that could be earned was 0, and the maximum was 20.

The questionnaire was pre-tested by researchers and lecturers at the Medical University of Gdańsk.

### 2.3. Statistical Analysis

The data were analyzed using Statistica v. 13.3 (TIBCO, Palo Alto, CA, USA), licensed to the Medical University of Gdańsk.

The sample size was calculated based on the estimated number of medical and dental students, derived from statistical data (population size *N* = 30,000, confidence level = 95%, margin of error e = 5%). A minimum of approximately 380 participants was required to obtain representative results.

The normal distribution of the analyzed variables was verified with the Shapiro–Wilk test. The descriptive statistics calculated included the number of observations (N), average (M), median (Me), minimum value (Min), maximum value (Max), and standard deviation (SD). In the analysis of relationships between features, the Pearson χ^2^ independence test and Fisher’s exact test were used. The reliability of the survey was analyzed. Student’s *t*-test was used to compare the overall test result against two independent groups (field of study). Pearson’s r correlation analysis was performed to examine the relationship between years of study. All tests were considered statistically significant at *p* ≤ 0.05.

## 3. Results

### 3.1. Characteristics of the Study Group

This study involved 562 participants. After excluding incomplete questionnaires, 541 remained and were included in the analysis. Twenty-one surveys were excluded due to substantial missing data. These individuals either did not answer most of the questions or responded only to those they were likely familiar with. The average age was 21 years (Min. = 16; Max. = 54; SD = 2.58). In the group of medical students, the ages ranged from 18 to 54 years, with a mean age of 21.6 (SD = 2.83). Among the dentistry students, the age ranged from 16 to 34 years, with a mean age of 21 (SD = 2.17).

Most of the study participants were women (40/562, 81.33%). They constituted 75.97% (234/308) of the group of medical students and 88.41% (206/233) of the dentistry students.

In total, 367 participants (67.84%) were in their first two years of study. The largest group of medical students were second-year students. The number of students in this group was 122 (39.61%). The smallest group, 18 people, included sixth-year medical students. Among the dentistry students, the majority, that is, 39.06% (n = 91), were first-year students. The homogeneity in the medical and dental student groups in terms of year of study was assessed using the χ^2^ test. The distribution of years differed significantly between the two groups, indicating that they were heterogeneous in this regard (*p* < 0.05).

A large city with a population of over 300,000 was the permanent place of residence of approximately half of the study participants (287/562, 53.05%). Significantly fewer students, only 33 (6.10%), lived in the countryside. Detailed information about the sociodemographic profiles of the students is presented in Table 1.

### 3.2. Comparison of Medical and Dental Students’ Knowledge About HPV

Analysis using the χ^2^ test revealed a statistically significant difference between the medical and dental students in terms of knowledge about the most oncogenic HPV subtypes. The medical students achieved significantly higher scores, with the percentage of correct answers reaching almost 40%, compared to 30% among the dental students. The medical students achieved better results on questions regarding the HPV abbreviation extension, its viral family, the most common HPV serotypes, and the age groups in which it is most prevalent. However, the dental students performed statistically better when asked whether HPV can cause oral cancer (Table 2).

The analysis of knowledge about oral cavity symptoms associated with HPV infection showed that the compared groups differed in their awareness of three out of the eight listed symptoms. Differences in knowledge about diseases caused by HPV were observed for 2 out of the 10 items. Regarding methods for treating oral lesions associated with HPV infection, the field of study influenced the results for three out of the eight reviewed treatments, with the medical students demonstrating a higher level of knowledge. No significant differences were found between the groups according to the frequency of correct answers to the remaining questions. When *p* > 0.05, we conclude that both groups performed similarly, providing correct answers with the same frequency (Table 3).

Both groups were analyzed for their overall HPV knowledge score. The mean total score in both groups was 11.35 points (Min. = 1; Max. = 19; SD = 3.30). In the group of medical students, the mean total score ranged from 1 to 19 points, with a mean score of 11.86 (SD = 3.33). In the dental student group, the mean total score ranged from 1 to 19 points, with a mean score of 10.64 (SD = 3.12).

Cronbach’s alpha test was conducted to assess the reliability of the survey, yielding a value of 0.87. This indicates that the individual survey items effectively measure the same construct. The result reflects a very good level of reliability.

**Figure 1:**
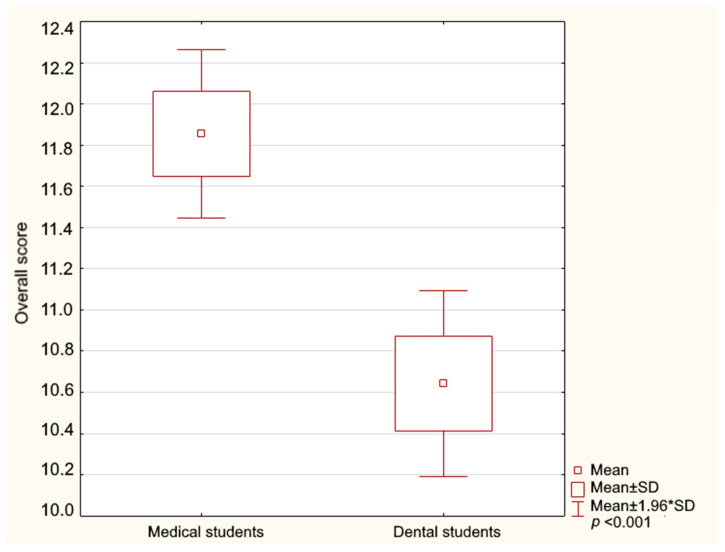
The overall result of the HPV knowledge test results among the medical and dental students (1.96*SD—standard deviation range).

A Student’s *t*-test was performed, revealing that the medical students achieved a significantly higher average score compared to the dental students. The medical students demonstrated a significantly higher level of knowledge on the topic (*t* = 3.87, df = 439, *p* < 0.001). The distribution of the overall score across the study group is illustrated in Figure 1.

### 3.3. Comparison of Groups Based on the Overall HPV Knowledge Score

The relationship between the respondents’ year of study and their level of knowledge about HPV was analyzed, revealing a statistically significant correlation. Higher years of study were associated with higher knowledge test scores. This trend was observed both for all the respondents combined (r = 0.48, *p* < 0.001) and separately among the medical (r = 0.5, *p* < 0.001) and dentistry students (r = 0.41, *p* < 0.001).

**Figure 2:**
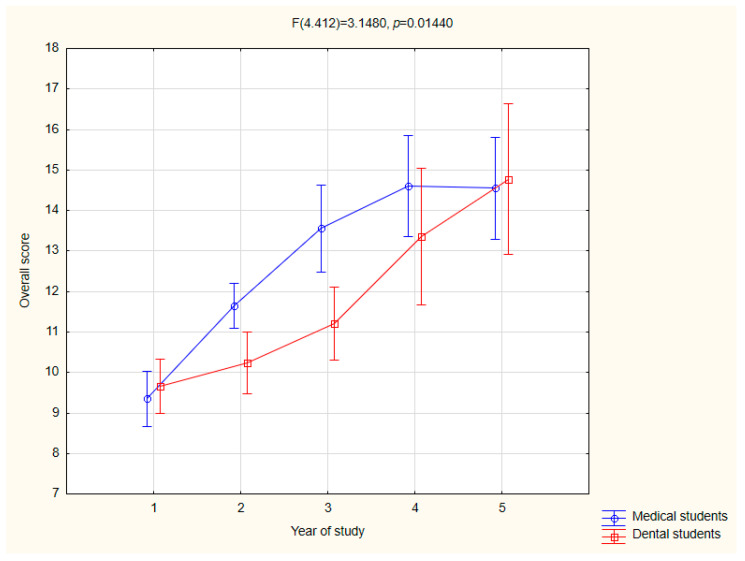
Graph of the relationship between the overall test result and the year of study.

The relationship between the respondents’ field and year of study and their level of knowledge about HPV was analyzed, revealing a statistically significant correlation (*p* < 0.05; Figure 2). Higher years of study were associated with higher knowledge test scores. This trend was observed both for all the respondents together (r = 0.48, *p* < 0.001) and separately among the medical students (r = 0.5, *p* < 0.001) and dentistry students (r = 0.41, *p* < 0.001). By the end of their education, the students in both fields demonstrated improved knowledge. The dental students achieved an average of 74% correct answers, while the medical students achieved 73%.

## 4. Discussion

Considering the widespread problem of HPV infection and its impact on the development of lesions on the skin and mucous membranes, future doctors should possess fundamental knowledge in this field [1,2,3,4,5,6,16,17,18,19]. Students had quite a good understanding of HPV, recognizing it as a DNA virus and knowing its routes of transmission. However, their knowledge of the virus family, as well as the most common and highly oncogenic serotypes, was significantly lower.

In our study, we focus on the problem of HPV infection and its impaction lesions in the head and neck area primarily. Warts are commonly observed on the skin as a consequence of HPV infection. They are generally asymptomatic. Patients may report white or pink spots, fluid-filled vesicles that can cause itching, discomfort, or irritation in the affected areas. On the mucous membrane, small painless growths may appear, along with changes in tissue color and consistency. HPV infection may not cause systemic symptoms. HPV is involved in the pathogenesis of some benign oral lesions. It leads to the formation of squamous cell papilloma, verruca vulgaris, and focal epithelial hyperplasia (FEH) [19,20,21,22,23,24]. These lesions may occur singly or multiply, anywhere in the oral cavity, but are most frequently found on the tongue, soft palate, and lips [21]. A standard diagnostic approach involves obtaining a sample from the lesion for histopathological examination to confirm its non-malignant nature [3,7,8].

Several studies have assessed the role of HPV in the development of potentially malignant oral disorders (PMODs). They mainly include leukoplakia, erythroplakia, oral lichen planus, or proliferative verrucous leucoplakia. Whether HPV infection influences the development of PMODs or has an adverse effect on the progression and prognosis of such lesions is still a matter of debate [21]. However, an association between infection with oncogenic HPV types 16 and 18 and the etiopathogenesis of oral lichen planus and oral leukoplakia has been confirmed [25].

The participants of our study stated that they knew the main symptoms of HPV infection and were aware of its role in the development of epidermodysplasia, genital warts, and oral cancer. What they did not know or did not know enough about was that HPV is responsible for the formation of white or red spots, leukoplakia, cauliflower-like eruptions, exophytic nodules, pink cauliflower-shaped nodules, and ulcerated tumors. The percentage of correct answers was below 50%.

Surgical excision with a cold-blade scalpel is the most commonly recommended treatment procedure for HPV-related oral lesions. An alternative to the traditional scalpel is the use of a laser. The goal of both techniques is a complete excision of a lesion, including its base and a margin of clearance around the base. Laser therapy is considered to be well-tolerated because of minimal postoperative pain and edema and lack of sutures. As for the operator, this technique also seems to be more advantageous, as it allows for high precision in tissue destruction, excellent hemostasis, and very good control of bleeding. However, both procedures are effective with a low rate of recurrence [26,27]. The other treatment options include cryotherapy, the use of antiproliferative drugs (podophyllotoxin, 5-fluorouracil), keratolytic agents (salicylic acid, trichloroacetic acid, dichloroacetic acid), and sinecatechins [28,29]. It is important to remember that in addition to the treatment of removing the lesion, special attention should be paid to identifying the cause of the HPV infection. Detection and elimination of any chronic mechanical irritation and patient education to avoid risky habits, such as cigarette smoking and alcohol consumption, should be a part of HPV infection therapy. As poor oral health is an independent risk factor for oral HPV infection, patients should be also advised to maintain accurate oral hygiene, which is crucial for oral health [30]. The study participants were familiar with treatment methods, such as surgical removal, cryotherapy, and laser therapy, while antiproliferative or keratolytic drugs were correctly identified only by 46% of the respondents.

Data from 2022 on cancer cases derived from the Polish National Cancer Registry revealed that lip, oral cavity, and pharynx cancers constituted 3.5% of all cancers in men and 1.5% in women. Their incidence (standardized to the European population, ESP 2013) among men was 18.87/100,000, while among women, it was 6.4/100,000 [31]. The standardized lip, oral cavity, and pharynx cancer mortality rate was 13.32 deaths per 100,000 population for men and 3.7 for women. Symptoms of head and neck cancer that are observed most frequently include ulcerated lesions with a central necrotic area surrounded by raised borders that do not heal within 14 days, thickening of the mucous membrane, sore throat, and hoarseness. Additional symptoms may include dysphagia, odynophagia, and otalgia [7,8]. Human-papillomavirus-related squamous cell carcinoma of the oral cavity and pharynx (HPV+ OPSCC) mainly occurs as a small, primary tumor with concomitant cervical lymphadenopathy. In some patients, only cervical lymphadenopathy may occur. It is located mainly on the base of the tongue, the floor of the mouth, and the tonsillar complex. It also involves other parts of the tongue, the buccal mucosa, the retromolar region, the maxillary and mandibular alveolar process, the soft and hard palate, the posterior wall of the pharynx, and the uvula [3,6]. In our study, only 12.05% of the medical students and 9.48% of the dental students responded correctly to the most frequent site of HPV-associated OPSCC. This indicates that a significant number of respondents were not aware of the most common location of HPV + OPSCC.

The primary diagnostic test involves obtaining a sample from the lesion for histopathological examination, which confirms the malignant nature of the lesion tumor, as well as its type and degree of histological differentiation [3,7,8]. All asymptomatic neck tumors should be evaluated using ultrasound with an assessment of cervical lymph nodes and fine-needle aspiration biopsy. In cases where uncertainty remains regarding the extent of the tumor, additional imaging tests such as computed tomography or magnetic resonance imaging of the head and neck are performed [3,7,8,32].

Clinical manifestations of HPV-related oral squamous cell carcinoma are similar to those caused by other carcinogens. There are some features, however, suggesting that the cancer may be HPV-positive. A key aspect of the carcinogenesis of HPV-related squamous cell carcinoma of the head and neck is that it occurs in younger age groups compared to tobacco-related cancer, has a better prognosis, and is more common in women than in men [6,7,8,9]. Most of the respondents correctly stated that this type of cancer occurs more frequently in women, but half of the medical students and only slightly more than one-third of the dental students knew that HPV-positive cancer is more common in patients before the age of 50. The participants’ knowledge about survival rates of HPV-related oral cancer was dramatically low, as less than 10% provided correct answers.

The choice of treatment method for HPV-related squamous cell carcinoma depends on the location and diameter of the primary tumor, the degree of differentiation (well, moderately, poorly differentiated, or undifferentiated), the presence of positive surgical margins, and the involvement of surrounding lymph nodes. Treatment is usually surgical. In many cases, it involves the excision of the tumor with a margin of healthy tissue, followed by immediate reconstruction. If the disease is advanced, surgical removal of the appropriate group of lymph nodes is recommended. Some minimally invasive surgical methods include transoral laser microsurgery and transoral robotic surgery. These methods are used when the primary tumor is not large (not exceeding T3 according to the TNM classification), when good transoral access is available, and when there is no bilateral lymph node involvement. Adjuvant therapy consists of postoperative radiotherapy or chemotherapy. The current standard radiotherapy dose is 66–70 Gy, while chemotherapy is typically based on cisplatin [3,6].

As with many aspects of health, prevention is the key and includes vaccinations and regular check-ups. Vaccination is highly effective and should be administered before the reproductive period for both girls and boys. According to sources, the nine-valent HPV vaccine theoretically reduces the potential for oral HPV carcinogenesis. It covers all subtypes of HPV occurring in the oral cavity, except for HPV 59, which is extremely rare [3,33,34]. The students were aware of the need for vaccinations and their recommendations for both women and men, as well as the fact that the Polish Vaccination Program recommends this vaccination (with the percentage of correct answers exceeding 78%). However, they did not know how many types of vaccines are available.

Studies investigating the awareness of HPV infection among dental and medical students are limited; therefore, some of the conducted research presents similar results to this study. A cross-sectional study conducted on 886 undergraduate dental students from Egypt, India, Pakistan, Saudi Arabia, UAE, and Sudan presented a poor knowledge of diseases caused by HPV, HPV vaccines, and the clinical appearance of the early lesion of oral cancer [35]. A cross-sectional study conducted among dental students, interns, and postgraduate maxillofacial residents at the University of Jordan presented that all groups had insufficient knowledge regarding the availability of the HPV vaccine and that most HPV infections resolve within a short period of time. Third- and fourth-year students showed superior knowledge of HPV compared to first- and second-year students [36]. In a cross-sectional study conducted by Murariu et al. [37] among dental students and residents, most of the participants stated that they did not have sufficient knowledge about the forms of oral cancer. Most of the participants were aware of the existence of an HPV vaccine; however, they were not aware of the possibility of preventing oral cancer by vaccination. Fifth-year students and residents had better knowledge of ways of HPV transmission, knew that HPV infection is asymptomatic, and identified the most common sites for oral cancer compared to fourth-year students [37]. A questionnaire evaluating HPV knowledge administered to first-year dental students at two Latin American universities revealed a lack of knowledge about the fact that most HPV infections spontaneously resolve, the age group for which HPV incidence is highest, the effectiveness of the HPV vaccine, and the proper time of vaccine administration [38]. A cross-sectional study conducted on dental students in Naples, Italy, presented a high knowledge of the correlation between HPV, OPSCC, and the HPV vaccine; however, an early-career group of students had less knowledge of the symptoms of the infection compared to late-career students. The participants of the study presented a good perception of their future role in preventing HPV infection; therefore, they reported gaining inadequate knowledge of HPV during their studies [39].

This study was limited by the heterogeneity in the study group. The students were from different academic years, fields of study, and universities, which could have influenced differences in their education programs. The main participants in this study were first- and second-year students, and these topics may not have been covered in their curriculum. Generally, the medical students had a higher level of knowledge, while among the participants in both fields of study in years 1 and 5, the results were similar. There was a clear increase in knowledge corresponding to the higher level of education (higher year of study), which is consistent with the expected learning curve. Medical and dental students should have extensive knowledge about the prevention, diagnosis, and treatment of HPV-related conditions, with particular emphasis on head and neck cancer as well as cervical cancer. Both medical and dental students discuss the topic of HPV in various subjects. In relation to the medical faculty, these subjects, among others, include gynecology, virology, gynecology, and oncology, and in the dental faculty, in addition to those mentioned earlier, periodontology and oral and maxillofacial surgery are included. That is why we decided to choose students from different years. The results may have differed between the dental and medical students because medical students typically receive more comprehensive education in infectious diseases—including HPV-related cancers, such as cervical cancer. In contrast, dental students focus more on oral diseases, so their knowledge of HPV may be more closely related to head and neck cancers. In our study, many of the questions addressed general knowledge about HPV, which may have resulted in more favorable outcomes for the medical students. It would be valuable to assess students’ knowledge after they complete their education. This represents a significant source of bias and a limitation of the current study. The strength of this study is that it included a large group of respondents, which makes this study representative.

Future directions of education could include seminars aimed at expanding knowledge on this specific topic. On the other hand, studies on knowledge assessment could focus on a cohort study comparing the initial knowledge of dental and medical students after their first year of study and then again after graduation. This would allow for a comparison of knowledge at the end of their studies and after several years of practice as a doctor. Social media campaigns have raised public awareness, which, together with recent changes in the vaccination policy in Poland, has led to an increase in vaccination rates among the population [10,11,12]. However, a rate below 20% is unsatisfactory. Therefore, educational meetings should be organized for teenagers and their parents to help them understand the importance of early prevention through vaccination for long-term protection against HPV in adulthood.

## 5. Conclusions

Medical students are characterized by a higher level of knowledge of the discussed topic than dental students. Both groups provided correct answers at a relatively unsatisfactory level because the overall value of the results obtained was below 60%. The Polish grading system in higher education is based on the 5 to 2 scale, where 5 is the best grade and 2 is the worst, meaning unsatisfactory. It denotes < 60% correct answers. A 60% score is sufficient to pass a knowledge test, but it indicates only a basic understanding of the material with room for improvement. In the context of our study, a score of 60% means that the respondents have some knowledge about HPV, but there are gaps in the comprehensive understanding of this health problem. To achieve advanced knowledge of the subject, it is necessary to deepen the knowledge of students, especially in the field of prevention, diagnosis, and the relationship of HPV with cancer. Concerning public health, it is important that physicians’ and dentists’ knowledge about HPV is comprehensive, especially since the virus has significant health consequences. Therefore, the quality of education at universities on the relationship between HPV infection and oral lesions and the importance of vaccination in the prevention of HPV-related diseases should be improved due to its great socio-economic importance.

## Figures and Tables

**Table 1:** Frequency distribution of sociodemographic profiles of the students.

Question	All		Medical Students		Dental Students	
	n	%	n	%	n	%
Gender						
Female	440	81.33	234	75.97	206	88.41
Male	101	18.67	74	24.03	27	11.59
Year of study						
I	178	32.9	87	28.25	91	39.06
II	189	34.94	122	39.61	67	28.76
III	79	14.6	32	10.39	47	20.17
IV	38	7.02	24	7.79	14	6.01
V	38	7.02	25	8.12	14	1.01
VI	19	3.51	18	5.84	0	0
Place of permanent residence						
Countryside	33	6.1	14	4.55	19	8.15
<100,000 inhabitants	118	21.81	62	20.13	56	24.03
100,000–300,000 inhabitants	103	19.04	66	21.43	37	15.88
>300,000 inhabitants	287	53.05	166	53.9	121	51.93

**Table 2:** Comparison of the medical and dental students regarding their level of knowledge about HPV with correct answers.

No	Survey	Groups		Statistics			
		Medical Students (%)	Dental Students (%)	χ^2^	*df*	*p*	*Fi*
1	HPV stands for *Human papillomavirus*.	97.08	93.56	3.87	1	0.049	−0.08
2	HPV belongs to *Papillomaviridae family*.	65.26	46.12	19.78	1	0	−0.19
3	HPV is a DNA virus.	57	51.93	1.38	1	0.241	−0.05
4	The most common HPV serotypes are 16, 18, 6, and 11.	54.22	34.33	21.14	1	0	−0.20
5	The routes of HPV infection include: sexual contact, vertical transmission, sharing towels or sponges.	80.19	78.11	0.35	1	0.554	−0.03
6	Highly oncogenic HPV serotypes associated with head and neck carcinomas are 16 and 18.	39.94	30.17	5.49	1	0.019	−0.10
7	The most common general symptom of HPV infection is usually asymptomatic.	93.81	95.28	0.54	1	0.461	0.03
8	HPV infection can contribute to the development of oral cancer.	85.71	91.34	4	1	0.046	0.09
9	Women are most at risk of developing cancer due to HPV infection.	68.83	63.52	1.68	1	0.195	−0.06
10	HPV-related oral cancer is more common in younger age groups (under 50 years old).	50	35.78	10.87	1	0.001	−0.14
11	HPV-related oral cancer most commonly affects the base of the tongue.	12.05	9.48	0.89	1	0.344	−0.04
12	The most common symptom of oral squamous cell carcinoma is an ulcer.	39.34	41.38	0.23	1	0.634	0.02
13	The 5-year survival rate for HPV–related oral cancer exceeds 50%.	8.82	7.33	0.39	1	0.531	−0.03
14	Orogenital contact increases the risk of oral cancer.	81.7	78.45	0.88	1	0.348	−0.04
15	HPV vaccination is recommended for both men and women.	81.05	78.88	0.39	1	0.533	−0.03
16	HPV vaccination is not mandatory in Poland.	92.18	88.79	1.8	1	0.179	−0.06
17	Two types of HPV vaccines are available.	14.33	16.88	0.66	1	0.417	0.03

(χ^2^—Pearson’s test value; *df*—degrees of freedom; *p*—level of statistical significance; *Fi*—measure of dependence strength; red font — statistically significant *p* ≤ 0.05).

**Table 3:** Comparison of the medical and dental students regarding their level of knowledge about HPV with correct answers (underlined)—multivariate questions.

HPV Infection May Contribute to the Development of the Following Diseases	Groups		Statistics			
	Medical Students (%)	Dental Students (%)	χ^2^	*df*	*p*	*Fi*
Epidermodysplasia	21.18	12.15	6.99	1	0.008	−0.12
Genital warts	63.79	43.11	22.23	1	0	−0.21
Hunter’s tumor	24.14	24.31	0	1	0.964	0
Leukoplakia	33.67	30.59	0.55	1	0.46	−0.03
Oral squamous cell carcinoma	78.19	75.55	0.51	1	0.475	−0.03
Oropharyngeal squamous cell carcinoma	70.37	63.84	2.49	1	0.115	−0.07
Papilloma	83	76.32	3.63	1	0.057	−0.08
Papillomatosis	63.76	63.27	0.01	1	0.909	0
Plummer–Vinson syndrome	25.09	20.18	1.69	1	0.193	−0.06
Warts	78.67	72.57	2.63	1	0.105	−0.07
HPV symptoms	Groups		Statistics			
	Medical students (%)	Dental students (%)	χ^2^	*df*	*p*	*Fi*
Cauliflower eruptions	56.44	55.66	0.03	1	0.859	−0.01
Exophytic nodules	43	37.95	1.36	1	0.244	−0.05
Hard ulcer	33	20.27	10.35	1	0.001	−0.14
Pink, cauliflower-shaped nodules	52	42.79	4.33	1	0.037	−0.09
Red and white spots	37.21	33.03	0.97	1	0.324	−0.04
Tumour with necrosis	34.55	22.52	8.9	1	0.003	−0.13
Ulceration	52.48	55.11	0.36	1	0.548	0.03
White spots	44.08	39.01	1.36	1	0.244	−0.05
Methods for treating oral lesions associated	Groups		Statistics			
with HPV infection	Medical students (%)	Dental students (%)	*χ^2^*	*df*	*p*	*Fi*
Cryotherapy and laser therapy	61.41	58.67	0.4	1	0.526	−0.03
Surgical excision	74.92	69.47	1.92	1	0.166	−0.06
Systemic antibiotic therapy	63.85	48.89	11.7	1	0.001	−0.15
Use of antiproliferative drugs (podophyllotoxin, 5-fluorouracil)	46.44	37.1	4.51	1	0.034	−0.09
Use of immunomodulators (interferon α, imiquimod)	9.15	10.67	0.33	1	0.565	0.03
Use of keratolytic agents (salicylic acid, trichloroacetic acid, dichloroacetic acid)	24.23	20.27	1.14	1	0.287	−0.05
Use of antiviral drugs (e.g., acyclovir)	21.55	14.67	4.01	1	0.045	−0.09
Use of sinecatechins	11.3	10.36	0.12	1	0.734	−0.01

(χ^2^—Pearson’s test value; *df*—degrees of freedom; *p*—level of statistical significance; *Fi*—measure of dependence strength; red font—statistically significant *p* ≤ 0.05).

## Data Availability

The data presented in this study are available on request from the corresponding author and Supplementary Materials. The data are not publicly available due to privacy restrictions.

## Supplementary Materials

The following supporting information can be downloaded at: https://www.mdpi.com/article/10.3390/jcm14082695/s1, Assessment of the awareness of medical and dental students about the impact of HPV infection on the development of oral cancers.
