# Supplementary material for: Evaluation of Knowledge of Human Papillomavirus Infection and Its Oral Health Implications: A Comparative Study of Polish Medical and Dental Students
Source: J Clin Med. 2025 Apr 15;14(8):2695. doi: 10.3390/jcm14082695 (PMC12028054; doi:10.3390/jcm14082695)
Supplement: Supplementary file 1 [file jcm-14-02695-s001.zip › jcm-3560711-supplementary.pdf]

Assessment of the awareness of medical and dental students about the impact of HPV infection on the development of oral cancers.

<u>Basic information</u>

Gender
Female
Male

Age – please fill in ………………………………..

Please select your field of study:
Medical
Dental

Permanent place of residence
Rural area
City up to 100 thousand inhabitants
City up to 300 thousand inhabitants
City over 300 thousand inhabitants

<u>Detailed information</u>

HPV is:
human papillomavirus
human herpes virus
varicella zoster virus
I don't know

HPV belongs to the family:
herpesviridae
papillomaviridae
retroviridae
I don't know

The HPV is a:
DNA virus
RNA virus
I don't know

The most common HPV serotypes are:
16, 18, 6, 11
31, 33, 56
51, 52, 56
I don't know

The routes of HPV transmisson are:
through sexual contact
vertical transmission – childbirth
using of the same towels, sponges
all correct
I don't know

Which HPV subtypes are highly oncogenic for head and neck cancers?
6, 11, 42, 43
32, 4, 33
5, 8, 14
16, 18
I don't know

May an individual be HPV infected and have no symptoms?
yes
no
I don’t know

Can HPV infections lead to oral cancer?
yes
no
I don't know

Which of the following diseases/lesions is/can be caused by the HPV?

I don’t know	No	Yes	Leukoplakia
I don’t know	No	Yes	Genital Warts
I don’t know	No	Yes	Hunter's tumor
I don’t know	No	Yes	Warts
I don’t know	No	Yes	Epidermodysplasia verruciformis
I don’t know	No	Yes	Gummas
I don’t know	No	Yes	Papilloma
I don’t know	No	Yes	Plummer–Vinson syndrome
I don’t know	No	Yes	Papillomatosis
I don’t know	No	Yes	Oral squamous cell carcinoma
I don’t know	No	Yes	Oropharyngeal squamous cell carcinoma

Symptoms of the oral cavity HPV infection include:

I don’t know	No	Yes	White spots
I don’t know	No	Yes	Red and white spots
I don’t know	N0	Yes	Cauliflower eruptions
I don’t know	No	Yes	Hard ulcer
I don’t know	No	Yes	Exophytic nodules
I don’t know	No	Yes	Pink, cauliflower-shaped nodules
I don’t know	No	Yes	Ulceration
I don’t know	N0	Yes	Tumor with necrosis

Which gender is more likely to develop cancer due to HPV infection?
male
female
it doesn't matter
I don't know

HPV-related oral cancer occurs in:
younger age groups (under 50)
older age groups (over 50)
I don't know

HPV-related oral cancer most often affects:
floor of the mouth
body of the tongue
base of the tongue
I don't know

The most common symptom of oral squamous cell carcinoma is:
difficulty swallowing and pain
ulceration
trismus
bleeding
I don't know

What is the 5-year survival rate for HPV-related head and neck cancers?
up to 5%
15-20%
4-50%
over 50%
I don't know

Does orogenital contacts increase the risk of oral cancer?
yes
no
I don't know

Methods for treating oral lesions associated with HPV infection include:

I don’t know	No	Yes	Systemic antibiotic therapy
I don’t know	No	Yes	Cryotherapy and laser therapy
I don’t know	No	Yes	Surgical excision
I don’t know	No	Yes	Use of immunomodulators (interferon α, imiquimod)
I don’t know	No	Yes	Use of antiviral drugs (e.g. acyclovir)
I don’t know	No	Yes	Use of antiproliferative drugs (podophyllotoxin, 5-fluorouracil)
I don’t know	No	Yes	Use of keratolytic agents (salicylic acid, trichloroacetic acid, dichloroacetic acid)
I don’t know	No	Yes	Use of sinecatechins

Who should get vaccinated against HPV?
only young girls
only women
only men
all correct
I don't know

Is the HPV vaccination mandatory?

yes

no

I don't know

How many types of HPV vaccines are there?

two

one

three

four

I don't know
